# Synergistic efficacy of Bisbenzimidazole and Carbonyl Cyanide 3-Chlorophenylhydrazone combination against MDR bacterial strains

**DOI:** 10.1038/srep44419

**Published:** 2017-03-17

**Authors:** Devapriya Sinha, Stuti Pandey, Raja Singh, Vinod Tiwari, Kirti Sad, Vibha Tandon

**Affiliations:** 1 Chemical Biology laboratory, Department of Chemistry, University of Delhi, Delhi, India; 2 Special Centre for Molecular Medicine, Jawaharlal Nehru University, New Delhi, India

## Abstract

Activation of efflux systems and the formation of biofilm are majorly adapted by microbes to resist antimicrobial agents. PPEF (bisbenzimidazole) targeting topoisomerase IA is observed to be an effective bactericidal agent against both Gram-positive and Gram-negative bacterial strains and thus can be developed as potent broad-spectrum antibiotic against MDR strains. PPEF treatment did not cause target specific mutation instead it leads to up-regulation of efflux gene in *E. coli* K12 as a mechanism of resistance. Microscopy, fluorescence spectroscopy and flow cytometry result demonstrate higher accumulation of PPEF in efflux gene deleted *E. coli* K12 mutants, and also suggest that Carbonyl Cyanide 3-Chlorophenylhydrazone (CCCP), resist the efflux of PPEF, and thus increases efficacy of PPEF. Herein, we report, PPEF and CCCP synergistically killed the persistent bacterial cells, which are not killed by PPEF alone. The above two compounds together inhibited biofilm formation, eradicate preformed biofilms and kills the biofilm cells of *P. aeruginosa*. PPEF and CCCP together reduced bacterial load of *E. coli* ATCC25922 by 6 log_10_ in neutropenic thigh infection model of balb/c mice. Present study suggests that combination therapy could be a promising antimicrobial strategy to handle MDR pathogenic strains.

The emergence of multi-drug resistant (MDR) bacterial strains and their rapid world-wide spread are a threat to human health^1^,^2^. This crisis is global, which has occurred due to the world-wide repeated and improper use of drugs^3^,^4^. Development of new broad-spectrum antibacterial agent with novel target and new approach is required to overcome this situation^5^. Antibiotic accumulation in Gram-negative bacteria is primarily influenced by two factors, membrane permeability and efflux activity^6^. Studies have recognized activation of efflux pumps as one of the major cause of resistance to many classes of antibiotics^7^,^8^. Another challenge is to kill the biofilm-associated cells which shows activated efflux and have specific stress responses that contribute to the occurrence of persister cells^9^,^10^,^11^,^12^. Combination therapy of antibiotics has been demonstrated in the clinic and is preferred as a design strategy. Synergistic interactions are advantageous since, the activity is enhanced and thus for a given amount of drug, they more effectively inhibit the growth of drug-sensitive pathogens^13^,^14^. Association of efflux mechanisms to antibiotic resistance, suggest efflux pump inhibitors (EPIs) as adjuvants could potentiate the activities of antibacterial agent. This hypothesis leads us to study the effect of bisbenzimidazole in combination with EPIs to target the MDR bacterial strains, the persistent population and the sessile cells forming biofilm.

Our group has identified bisbenzimidazoles (BBZs) as a specific topoisomerase IA poison inhibitors which do not inhibit gyrase, human topoisomerase IB and human topoisomerase II enzymes^15^. Bacterial topoisomerase IA is a novel drug target and inhibitors developed to target the cleavage religation equilibrium of the catalytic activity of this enzyme are believed to be bactericidal^16^. We have demonstrated 2-(3,4-dimethoxyphenyl)-5-[5-(4-methylpiperazin-1-yl)-1H-benzimidazol-2-yl]-1H-benz-imidaz-ole (DMA) and 2′-(4-propyl piperazine-1-yl)-1H, 3′H-2,5′-bibenzimidazole (PPEF) as potent *E. coli* topoisomerase IA poison inhibitor^15^. Our study shows PPEF lead to Mg^2+^ chelation which is required by the topoisomerase IA for religation of the cleaved DNA and thus acts as bactericidal agent^17^.

The current study, demonstrate the antibacterial potency of BBZs against nosocomial pathogens *E. faecium, S. aureus, K. planticola, A. baumannii, P. aeruginosa* and *Enterobacter* sp. often referred as the ‘ESKAPE bugs’ which are known for extensive multidrug resistance^18^,^19^,^20^. Herein, the effect of PPEF on efflux pumps were studied in order to address resistant strains. Moreover, an important aspect of synergism between PPEF and efflux pump inhibitor CCCP was studied and the effect of these molecules in combination and individually on the persistent population, the sessile cells and their vivo efficacy were carried out.

In the present context we aimed to study the antibacterial effect of BBZs and compared its efficacy in the presence of efflux pump inhibitor CCCP. Herein, an important aspect of synergism between PPEF and CCCP has been demonstratedand validated by the different *in vitro* and *in vivo* studies.

## Results

### Antibacterial activity of BBZs against MDR strains

In the present study, 6 potent BBZs were assessed for their antibacterial activity against the common MDR human pathogenic strains *Klebsiella* sp., *A. baumannii, S. typhimurium, Enterococcus* sp., *S. aureus, S. flexineri, P. aeruginosa, Enterobacter* sp. and *Providencia* sp. (Tables 1 and 2). The drug resistance profiling of all the collected bacterial strains were determined as per CLSI guidelines and observed most of them as MDR strains (Supplementary Tables S1 and S2)^21^. The results indicates, that BBZs used in the study are not specifically targeting Gram-negative bacteria but also show significantly good antibacterial activity against Gram-positive bacterial strains which include pathogenic *Enterococcus* sp., *S. aureus* and the Methicillin-resistant Staphylococcus aureus (MRSA) strains (Table 2). Further, these six BBZs were observed to be bactericidal in nature (Supplementary Tables S3 and S4). The MIC values suggest PPEF as the most potent broad spectrum antibacterial agent among the six compounds used in the study.

### *De novo* generated resistant mutant of *E. coli* K12 against PPEF shows activated efflux as the mechanism of resistance

The present study was aimed to investigate whether resistant mutations against PPEF can appear *de novo* and take over the normal bacterial population or not. And further, if the resistant mutants were achieved, then to isolate the resistant mutants for characterization. For *de novo* generation of mutant, *E. coli* K12 cells were challenged with the sub-lethal dose of PPEF with gradual increment in the dose and after ~900 generations population of resistant *E. coli* K12 cells were achieved that could resist 64 μg/mL of PPEF which is equivalent to 8XMIC of the sensitive *E. coli* K12 (MIC 8 μg/mL) strains (Supplementary Table S5).

**Figure 1:**
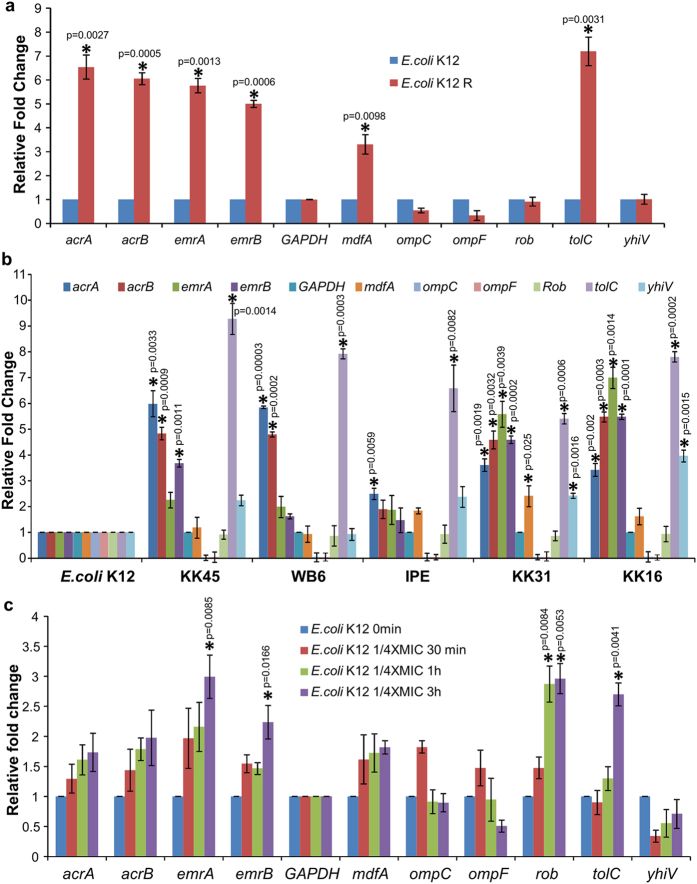
Resistant phenotypes show up-regulated efflux pumps and down-regulated porins. Relative gene expression analysis (**a**) *E. coli* K12 (control) and *de novo* generated PPEF resistant *E. coli* K12 measured by qPCR. (**b**) *E. coli* K12 (control) and MDR *E. coli* isolates (**c**) *E. coli* K12 (control) and *E. coli* K12 treated with ¼ XMIC PPEF for 30 mins, 1 h and 3 h. Results are presented as mean ± SD of three independent experiments (n = 3). Statistical comparison between control and experimental conditions were found to be statistically significant with *p < 0.01. Detailed statistically significant p-values of control *vs*. experimental are indicated.

To decipher the mechanism of the resistance so developed in this strain; the PPEF resistant *E. coli* K12 cells were studied for the target specific topoisomerase IA mutation a strategy commonly adapted by bacteria to develop resistance against specific antibiotic. However, we did not observe any mutation in the topoisomerase IA gene sequence including the region covering the three promoters present upstream of topoisomerase IA gene in the PPEF resistant *E. coli* K12 strains. Further to investigate the cause of resistance the changes in the relative gene expression responsible for efflux, influx and stress were studied. The resistant *E. coli* K12 showed up-regulation of efflux genes *tolC, acrA, acrB, emrA, emrB* and *mdfA* and down-regulation of porins *ompC* and *ompF* as compared to the untreated *E. coli* K12 strain (control) (Fig. 1a). Further, it is also observed that the MDR *E. coli* strains showing higher MIC values against PPEF (Supplementary Table S6) show over-expressed genes involved in efflux (Fig. 1b) probably a mechanism adapted by them to resist drugs. Treatment of sub-lethal dose of PPEF (1/4XMIC) showed significant increase in the efflux gene expression. Our result shows three hour exposure to PPEF increases the expression of efflux genes significantly accompanied by down-regulation of porin genes (Fig. 1c). Ciprofloxacin treated *E. coli* K12 cells were also observed to show significant up regulation of efflux gene *acrA, acrB* and *tolC* (Supplementary Fig. S1). Our result suggests PPEF is subjected to MDR efflux pump as most of the genes responsible for efflux were activated which was also observed in case of ciprofloxacin treated *E. coli* K12 cells.

### PPEF is subjected to efflux pumps

**Figure 2:**
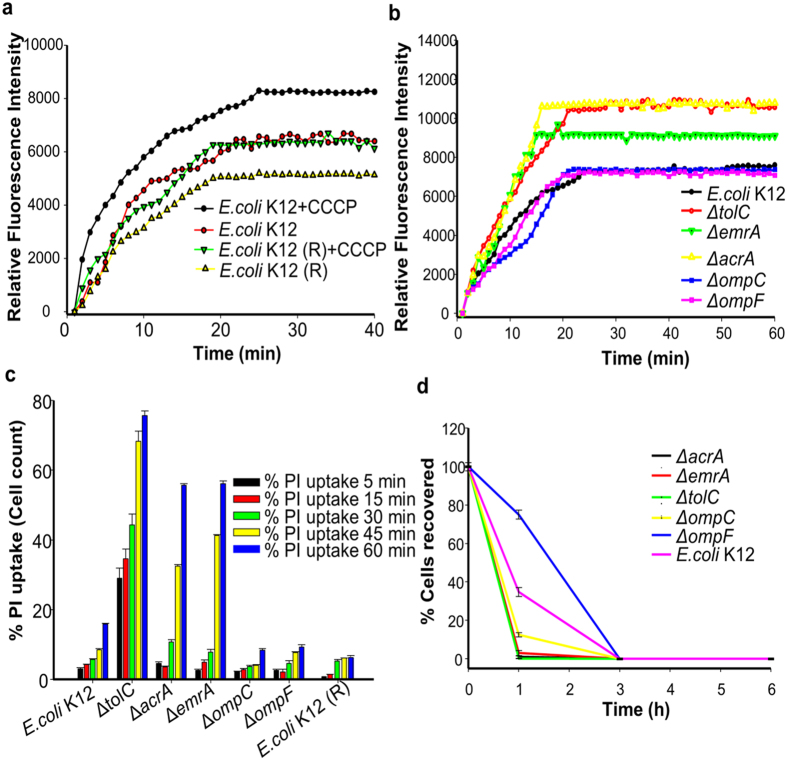
PPEF is subjected to efflux pumps. Time dependent analysis of accumulation of PPEF and bacterial cell death by fluorometric analysis, flow-cytometry and Time kill assay. (**a**) Accumulation of PPEF by *E. coli* K12 and *de novo* generated PPEF resistant *E. coli* K12 in presence and absence of CCCP over a period of 30 min incubation period. (**b**) Accumulation of PPEF in *E. coli* K12 & Δ*tolC*, ΔompC, Δ*acrA*, Δ*emrA* and Δomp F derived *E. coli* K12 mutants. (**c**) Percentage cell count of PI uptake quantified in *E. coli* K12 and other derived mutants treated with PPEF for the time points 5, 15, 30, 45 and 60 min by flow cytometer. Results are presented in mean (±SD) of three independent experiments (n = 3). (**d**) Percentage (%) of CFU recovered for *E. coli* K12 and derived mutants following treatment with PPEF. These data represent the result of mean (±SD) of three independent experiments (n = 3).

To further validate our observation, we investigated the accumulation kinetics of PPEF in *E. coli* K12 cells and the PPEF resistant *E. coli* K12 cells in presence and absence of efflux pump inhibitor (CCCP). Fluorescence spectroscopy was used to determine the accumulation kinetics as PPEF is fluorescent active molecule and hence on binding with DNA shows significant high fluorescent intensity denoting higher accumulation of PPEF in bacterial cells. As per our result, the *de novo* generated PPEF resistant *E. coli* K12 showed lower accumulation of PPEF as compared to the untreated *E. coli* K12 cells. In addition, when efflux pump inhibitor CCCP was used in combination, the accumulation of PPEF was more in the cells (Fig. 2a). *E. coli* K12 derived efflux deleted mutants Δ*tolC*, Δ*acrA* and Δ*emrA* also showed significant higher accumulation of PPEF in the cells (Fig. 2b).

**Figure 3:**
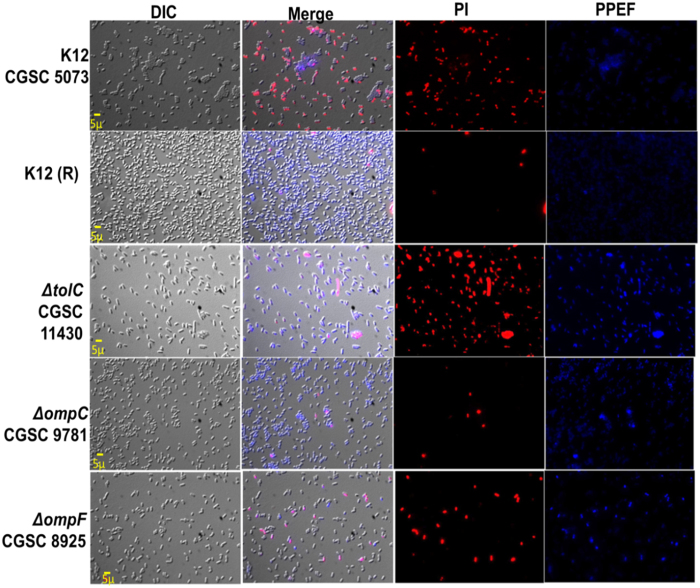
Analysis of PPEF uptake and PI staining using Zeiss microscope. Representative images of *E. coli* K12 and *E. coli* K12 derived (Δ*tolC*, Δ*ompC* and Δ*ompF*) mutants detected at emission wavelength range 450–500 nm for compound PPEF signal, 610–750 nm for PI signal and merged images are shown. Bacteria exhibiting blue fluorescence indicate PPEF uptake and red or purple fluorescence indicate damaged membrane and are considered as dead cells.

Further, we monitored the cell death kinetics by determining propidium iodide (PI) uptake by the cells treated with 1XMIC of PPEF for different time points 0, 5, 15, 30, 45 and 60 min by flow cytometry. Significant PI uptake shows higher bactericidal activity which was further confirmed by counting the colonies by plating the samples. Efflux deleted *E. coli* K12 mutants Δ*tolC*, Δ*acrA* and Δ*emrA* showed higher PI uptake whereas the PPEF resistant *E. coli* K12 cells showed lower PI uptake (Fig. 2c). Time kill assay was performed to validate the kinetics of killing of *E. coli* K12 and *E. coli* K12 derived (Δ*acrA*, Δ*emrA*, Δ*tolC*, Δ*ompC* and Δ*ompF*) mutants which was followed for 24 h. In case of PPEF treated *E. coli* K12 cells, 0% viable cells were recovered after 3 h treatment, whereas the efflux gene deleted *E. coli* K12 derived mutants Δ*tolC*, Δ*acrA* and Δ*emrA* showed 0 % cell recovery within 1 h of PPEF treatment. However, in case of PPEF resistant *E. coli* K12 cells, 0 % cell recovery of the viable cells was observed 12 h post-treatment (Fig. 2d). Fluorescence microscopy images confirm higher accumulation of PPEF leading to higher cell death. PPEF treated *E. coli* K12 and its derived mutants exhibited blue fluorescence whereas the dead cells exhibited red fluorescence with PI counter-staining. In the merged image the cells that are observed purple in colour shows uptake of both PPEF and PI. Cells those are stained by PI are considered dead and were further confirmed by plating the samples. Our result shows significant higher accumulation of PPEF in the Δ*tolC E. coli* K12 mutants leading to higher cell death, however lower intensity of the blue fluorescence due to PPEF uptake in the *de novo* generated PPEF resistant *E. coli* K12 strains suggest active efflux and hence lower cell death in this strain (Fig. 3).

### PPEF in combination with CCCP shows synergistic/additive effect against most of the bacterial strains

The *in vitro* effects of PPEF with CCCP in combinations were tested using the checkerboard dilution method. Among the combinations, we observed synergistic/additive effect against most of the bacterial strains used in this study (Table 3). PPEF used in combination with efflux pump inhibitor CCCP were observed to show >4 fold reduction in MIC against most of the pathogenic bacterial strains.

### Combination therapy kills the subpopulation of persistent cells and inhibits biofilm formation

**Figure 4:**
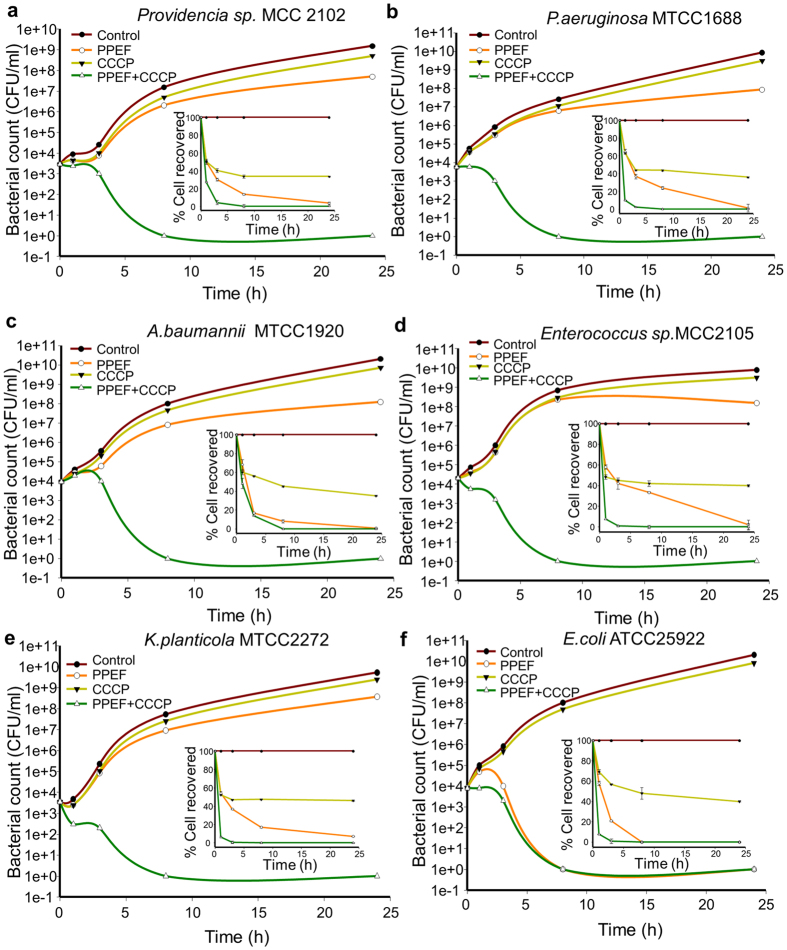
Combination of PPEF and CCCP kills the persistent population. Time-kill curves for bacteria with inset percentage (%) CFU recovered following treatment with 1XMIC PPEF, ½XMIC CCCP and 1XMIC PPEF + ½XMIC CCCP in combination. At the specific time intervals of post treatment viable cells were enumerated using plate count method. (**a**) *Providencia* sp. MCC2102 (**b**) *Pseudomonas aeruginosa* MTCC1688 (**c**) *Acinetobacter baumannii* MTCC1920 (**d**) *Enterococcus* sp. MTCC2105 (**e**) *Klebsiella planticola* MTCC2272 (**f**) *E. coli* ATCC25922. Inset graph shows results of % CFU recovered represent mean (±SD) of three independent experiments (n = 3).

The kinetics of cell killing is an important parameter to understand the efficacy of antimicrobial agents. Here, the killing activities of PPEF, CCCP alone and combination of PPEF and CCCP against the highly resistant bacterial strains *Providencia* sp. (MCC2102), *A. baumannii* (MTCC1920), *P. aeruginosa* (MTCC1688), *K. planticola* (MTCC2272), *E. coli* (ATCC25922) and *Enterococcus* sp. (MCC2105) was studied at a cell density of 10^4^ CFU/ml (Fig. 4). Both PPEF and CCCP exhibited bactericidal effect against the strains tested. We observed a 3 log_10_ reduction in the colony count for all the bacterial strains at 24 h when treated individually with PPEF and CCCP. However, in combination the effect was >5log_10_ reduction within 8 h of post treatment. The percentage of cell recovered after the above treatment was determined for each strainfrom the time kill curve. We observed substantial reductions in percent viability of the bacterial strains treated with PPEF, CCCP and PPEF, CCCP together (Fig. 4). Except *A. baumannii* (MTCC1920) all the bacterial species used in the study, showed less than 30 % cell recovery within 1 h, when treated with PPEF and CCCP together. However, >50% bacterial cells were recovered when treated with PPEF and CCCP individually for 1 h. We observed ~0% cell recovery after 3 h treatment when PPEF was used in combination with CCCP in most of the cases except for *A. baumannii* (MTCC 1920). In case of *A. baumannii* (MTCC 1920), 0% cell recovery was observed at 8 h treatment. The individual treatment with PPEF showed survival of sub-population of persistent cells (Fig. 4, graph inset). But, in case of combination 0 % cell were recovered after 8 h treatment suggesting combination of PPEF and CCCP target the persistent population of bacteria.

**Figure 5:**
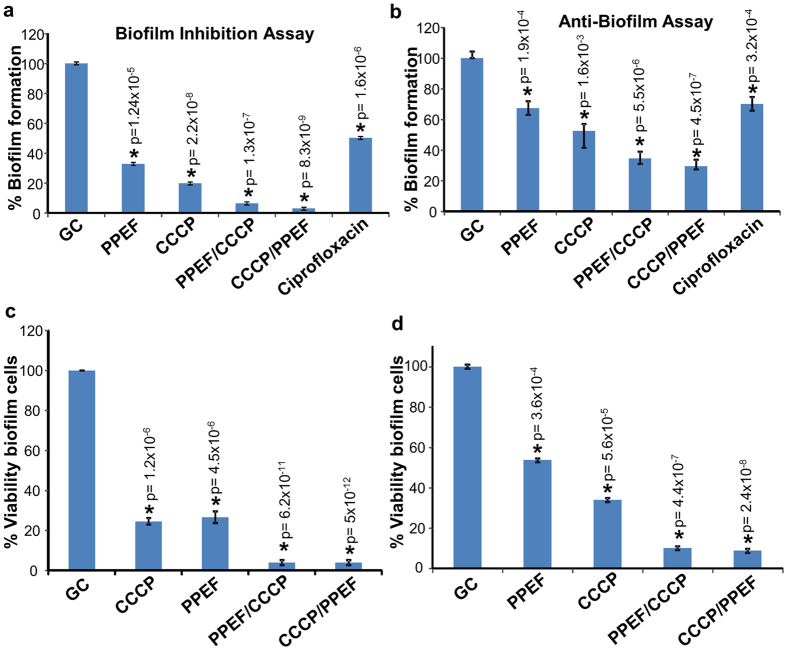
PPEF and CCCP in combination inhibit the biofilm mass, eradicate preformed biofilm and kills the biofilm forming cells. (**a**) Biofilm inhibition assay in presence (1XMIC) PPEF, (1XMIC) CCCP, in combination of (1XMIC) PPEF and (1/2XMIC) CCCP, in combination of (1XMIC) CCCP and (1/2XMIC) PPEF and 1XMIC ciprofloxacin. (**b**) Pre-formed biofilm eradication assay in presence of (1XMIC) PPEF, (1XMIC) CCCP, in combination of (1XMIC) PPEF and (1/2XMIC) CCCP, in combination of (1XMIC) CCCP and (1/2XMIC) PPEF and 1XMIC ciprofloxacin. (**c**) The percentage of viable cells forming biofilm determined by MTT with respect to control in presence (1XMIC) PPEF, (1XMIC) CCCP, in combination of (1XMIC) PPEF and (1/2XMIC) CCCP, in combination of (1XMIC) CCCP and (1/2XMIC) PPEF. (**d**) The percentage of viable cells treated after biofilm formation determined by MTT reduction to its insoluble formazan with respect to control samples in presence (1XMIC) PPEF, (1XMIC) CCCP, in combination of (1XMIC) PPEF and (1/2XMIC) CCCP, in combination of (1XMIC) CCCP and (1/2XMIC) PPEF. Results are presented of three independent experiments (n = 3) presented as mean ± SD. Statistical analysis was done by one way ANOVA followed by two tailed‘t’ test. Asterisks (*) indicates statistical significance and (*P ≤ 0.05) values are considered significant. Detailed statistically significant p-values of control *vs*. experimental are indicated.

The synergistic composition was also evaluated for the anti-biofilm assay against *P. aeruginosa* MTCC1688. Quantification of biofilm biomass indicated that PPEF and CCCP combination were superior to inhibit the biofilm formation and disrupt preformed biofilms than individually PPEF or CCCP (Fig. 5a and b). In the present study, we have also determined the percentage of viable biofilm cells through MTT assay. The PPEF and CCCP in combination showed significant killing (90–95 %) of biofilm cells as compared to the untreated control (Fig. 5c and d).

### *In vitro* toxicity of individual and combined treatment of PPEF and CCCP against HEK293T and NIH/3T3 cell lines

**Figure 6:**
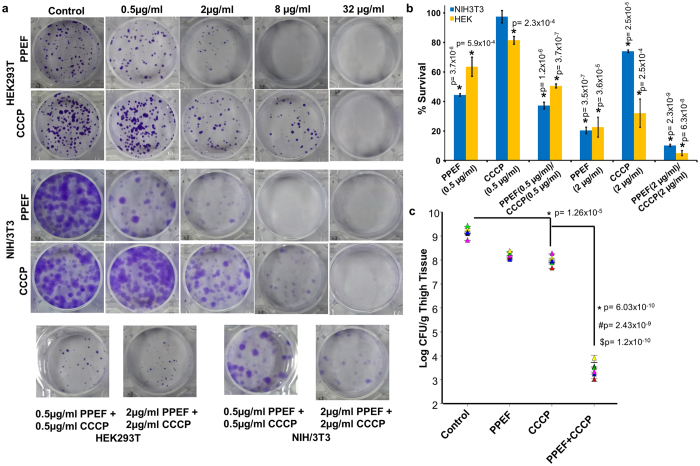
*In vitro* and *In vivo* efficacy of PPEF, CCCP and combination therapy. (**a**) Representative image of plates showing stained colonies treated with PPEF and CCCP alone and in combination for 24 h. (**b**) % Cell viability of NIH/3T3 and HEK293T cells in presence of indicated concentrations of PPEF, CCCP and PPEF + CCCP. Experiments are presented as mean ± SD (n = 3). Statistical analysis was done by one way ANOVA followed by two tailed ‘t’ test. Asterisks (*) indicates statistical significance and (*P ≤ 0.05) values are considered significant. Detailed statistically significant p-values of control *vs*. experimental are indicated. (**C**) *In vivo* efficacy of PPEF 3 mg/kg.bw, CCCP 3 mg/kg.bw and PPEF 3 mg/kg.bw + CCCP 3 mg/kg.bw in mice neutropenic thigh infection model. Graph represents Log_10_ CFU/g thigh *vs* drug dose in Balb/c mice. Asterisks (*) indicates statistical significance and (*P ≤ 0.05) with n = 6 animals in each group. Detailed statistically significant p-values are described below: control vs PPEF 0.8204, control vs CCCP 0.0000126, control vs PPEF + CCCP 0.000000000603, CCCP vs PPEF + CCCP 0.00000000243, PPEF vs PPEF + CCCP 0.000000000120.

The transformed human embryonic kidney cell (HEK-293T) and mouse embryonic fibroblast cell (NIH/3T3) were chosen for the study. We subjected both the cell lines for cell survival assay at concentrations 0, 0.5, 2, 8 and 32 μg/mL for 24 h and observed a dose dependent cytotoxicity (Fig. 6a). The treatment of NIH/3T3 cell with 0.5and 2 μg/mL of PPEF and CCCP each showed 44, 97 and 20, 74 % cell survival respectively. However, for HEK293-T, 63, 81 and 22, 32 % of cell survival was observed for PPEF and CCCP respectively. Further, to evaluate the combined *in vitro* cytotoxicity of PPEF and CCCP, two dosesof PPEF + CCCP {(0.5 + 0.5 μg/mL) and (2 + 2 μg/mL)} were chosen. We observed, 50 and 5 % viable cells for HEK293T and 37 and 10 % for NIH/3T3 respectively using above mentioned combined dose of PPEF and CCCP (Fig. 6b).

### *In vivo* efficacy validates combination therapy of PPEF and CCCP in Neutropenic Thigh Infection Model in Balb/c mice

The promising results of the *in vitro* studies, led us to investigate the efficacy of the synergistic composition of PPEF and CCCP in *in vivo*. PPEF was more potent than ciprofloxacin against *E. coli* ATCC25922 in neutropenic thigh infection model (Supplementary Fig. S4). The synergistic composition was tested in neutropenia thigh model of infection with *E. coli* ATCC25922. In this study, we have used same dosage of 3 mg/kg.bw each of CCCP and PPEF^21^. In both the cases we observed 1 log reduction in the bacterial load. However, when we used 3 mg/kg.bw of PPEF in combination with 3 mg/kg.bw of CCCP, we observed 6 log_10_ reduction in the bacterial count (Fig. 6c). The developed model validates the enhanced antibacterial activity of combination therapy.

## Discussion

**Figure 7:**
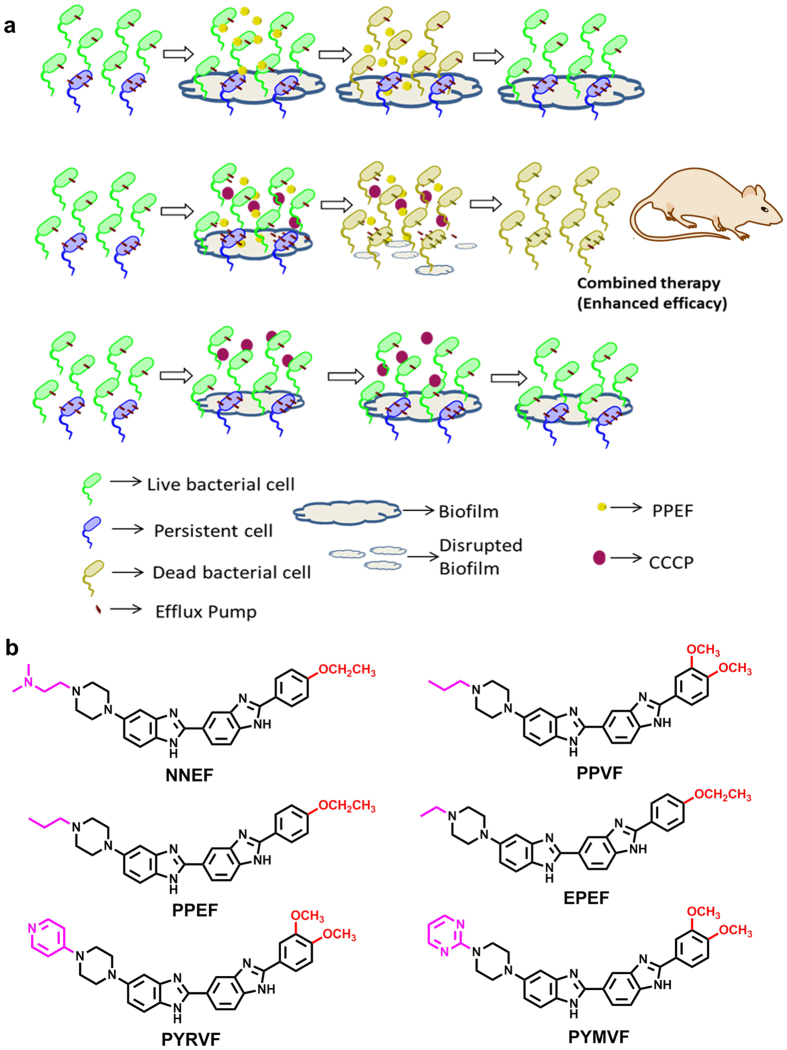
(**a**) Schematic presentation of enhanced antibacterial activity and efficacy by PPEF and CCCP combination therapy. (**b**) Chemical structures of Bisbenzimidazoles.

In our previous study, we have screened 24 BBZs for their antibacterial activity against water borne and clinical *E. coli* isolates collected from UTI patient samples and observed six compounds namely PPEF, PPVF, NNEF, PYRVF, PYMVF and EPEF structures depicted in (Fig. 7) to be the most potent^17^. These six compounds were also observed to be potent poison inhibitor of *E. coli* topoisomerase IA. Hence, we decided to further screen these six compounds against common broad spectrum pathogenic bacterial strains. The results of antibacterial activity showed PPEF as the most potent broad-spectrum antibacterial agent. Our result suggests, the propyl chain at the piperazine end of PPEF confers hydrophobicity to the molecule required for penetrating the LPS barrier of the bacterial cell membrane. On the other hand, the para-ethoxy group on phenyl ring of PPEF contributes to the increased cationic nature of the compound which helps the compound to interact with negatively charged cell wall of the bacterium. Few strains such as *A. baumannii, Klebsiella* spp. and *P. aeruginosa* show comparative higher MIC of these compounds. Report shows these strains have lower outer membrane permeability and higher exclusion limit suggesting the cause of resistance^22^. In the present study, we attempted to understand, how PPEF could contribute to generate mutants. We did not achieve any mutants of *S. aureus* and *E. coli* resistant to PPEF when treated the strains with 2X, 5X and 10XMIC of PPEF (Supplementary Fig. S2). But, with the serial passage of *E. coli* K12 strain with sub-MIC dose of PPEF, a population of *E. coli* K12 cells was achieved which could resist ~8XMIC of PPEF compared with the untreated one. However, the PPEF resistant *E. coli* K12 cells did not show any target specific topoisomerase IA mutation. But, these cells were observed to over-express efflux genes, which were also observed in the MDR *E. coli* strains. Further sub-MIC dose exposure also lead to significant up-regulation in the efflux gene expression suggesting PPEF is subjected to efflux. The bacterial efflux systems MFS, SMR, RND and MATE require transmembrane proton-motive force (PMF) for the electrochemical gradient to energize the export of drugs out of the cell^23^. CCCP acts as a protonophore which causes reduction of transmembrane potential required to be maintained for the efflux activity. Since, CCCP acts as chemical inhibitors of oxidative phosphorylation, which, in turn, serves to inhibit the activity of ATP synthase; therefore, CCCP have an effect on ABC superfamily pumps also which requires ATP for efflux activity^24^. Previous reports suggest that, CCCP disperse the membrane proton motive force by modifying the transmembrane electrochemical potential causing high toxicity to the cell^25^,^26^. When the efflux activity of the bacteria was challenged by an efflux pump inhibitor CCCP, an increased accumulation of PPEF and enhanced cell death was observed. Similar result was also observed in case of efflux deleted *E. coli* 12 derived mutants Δ*acrA*, Δ*emrA* and Δ*tolC* suggesting inactivation of efflux enhance the antibacterial efficacy of PPEF (Supplementary Table S7). Our result demonstrates PPEF in combination with CCCP shows synergistic effect against most of the MDR pathogenic bacterial strains (Fig. 7). This synergistic composition was also observed to kill the persisters which grow to the upper limit of mutation selection window^27^,^28^,^29^,^30^. This persister population were not killed by PPEF alone and thus probably could contribute in developing resistance. Some of the strains such as *A. baumannii, P. aeruginosa* and *S. aureus* were observed to form biofilms in order to resist against antibacterial agent. In the present study we demonstrate that the synergistic composition could also target biofilm formation and can eradicate preformed biofilm by *P. aeruginosa* (MTCC1688). In addition, both the drugs together could significantly kill the biofilm forming cells. Our earlier report demonstrates efficacy, of PPEF in Balb/c mice, which shows treatment of *E. coli* ATCC25922 infected mice with 5 mg/kg.bw of PPEF led to the 50 % of survival of the mice suggesting an ED_50_ of 5 mg/kg.bw, further 5 mg/kg.bw of PPEF was observed to show 3 log_10_ reduction in the bacterial count in neutropenic thigh infection model. In the present study, thigh infection model validates combination therapy of PPEF and CCCP is highly efficacious in mice infected with *E. coli* ATCC25922 with a significant 6 log_10_ reduction in the bacterial colony forming units.

## Conclusions

Present study reports the antibacterial activity of BBZs against both Gram-negative and Gram-positive bacteria. PPEF was observed to be the most potent antibacterial agent. Further, PPEF do not lead to target specific topoisomerase IA mutation but are subjected to efflux. Result shows blocking the efflux pump by CCCP enhanced antibacterial efficacy of PPEF against MDR bacterial strain. Both the compounds together kill the persistent population and bacterial cells with activated efflux. The synergistic composition was observed to inhibit biofilm formation and could eradicate preformed biofilm by *P. aeruginosa*. Moreover, PPEF and CCCP together could significantly kill the biofilm cells. Enhanced *in vivo* efficacy of the combination therapy suggests that strategy of using BBZ in combination with efflux pumpinhibitors may be used to develop antibacterial agents against the deadly MDR bacterial strains.

## Methods

### Materials

*E. coli* (ATCC25922) was procured from Himedia ltd, *Acinetobacter baumannii* (MTCC1920), *Pseudomonas aeruginosa* (MTCC1688), *Klebsiella planticola* (MTCC2272), *Salmonella typhimurium* (MTCC1251), *Shigella flexneri* (MTCC1457) were procured from CSIR-IMTECH, Chandigarh and *Enterococcus* sp. (MCC2105) was procured from NCCS, Pune. The MDR clinical Gram-positive strains *Staphylococcus* sp. (S1016 and S976) were provided by Dr. Rajni Gaind, Department of Microbiology, Vardhman Mahavir Medical College, New Delhi. MDR clinical Gram-negative strains *A. baumannii* (AB387), *P. aeruginosa* (PS162), *Klebsiella* sp.(K1164), *S. typhimurium* (ST412) were obtained from Institute of Pathology, Safdarjung Hospital, New Delhi, a national facility of Govt. of India. Dr. S. Chowdhury, CSIR-IGIB, New Delhi gifted us *S. aureus* MCC 740 and MRSA ATCC43300 strains for our study. *E. coli* K12 derived mutants Δ*ompC* (CGSC 9781), Δ*ompF* (CGSC 8925), Δ*emrA* (CGSC 10098), Δ*acrA* (CGSC 11843), Δ*tolC* (CGSC 11430) and *E. coli* K12 (CGSC 5073) was obtained from *E. coli* Genetic Stock Center, Yale University, USA. Antibiotics (ampicillin, kanamycin, chloramphenicol, ciprofloxacin, gentamycin, nalidixic acid, tetracycline, trimethoprim, and streptomycin) were purchased from Sigma and Mueller Hinton Broth and Agar were purchased from Hi Media. Balb/c mice were purchased from National Institute of Nutrition, Hyderabad. Animals were maintained under controlled conditions with free access to food and water in the animal house facility of Jawaharlal Nehru University. All animal experiments were approved by IAEC of Jawaharlal Nehru University, New Delhi, India performed using ethical and ARRIVE guidelines.

### Antibacterial Susceptibility Test

Minimal Inhibitory Concentration (MIC) and Minimal Bactericidal Concentration (MBC) were determined as recommended in CLSI guidelines^31^. The bacterial suspensions of 1.0 × 10^6^ CFU per/well were seeded in 96-well plates (Corning^®^ 96-well Clear Polystyrene Microplates) in presence of BBZ and antibiotics used in the study at concentrations 0.25, 0.5, 1, 2, 4, 8, 16, 32, 64, 128 μg/mL and incubated at 37 °C for 24 h. MIC values were scored as the minimal concentration at which no visible growth of bacterium were observed and were detected by Tecan Micro-plate Reader at 600 nm.

MBC values were determined by plating 100 μL of each clear well from the MIC micro-broth plate that had been incubated for 24 h at 37 °C on Mueller - Hinton agar plates without antibacterial compound for overnight at 37 °C. MBC endpoints were defined as the lowest dilution of compound which resulted in 99.9 % killing of the bacterial cells from the starting inoculum^32^.

### *De novo* generation of resistant mutants

PPEF resistant *E. coli* K12 mutants were generated as per previous method with minor changes as described below^33^. Three lineages were started from overnight cultures from independent colonies, using an initial number of ~10^4^ *E. coli* K12 cells. The cells were serially passaged by 1000-fold dilution in 1 ml batch cultures every 24 h for 900 generations in Mueller-Hinton medium containing PPEF. Subsets of these cells were restreaked on Mueller-Hinton agar containing PPEF of same concentration to confirm that they were resistant. After 900 generations, a population of *E. coli* K12 cells were achieved that could resist 64 μg/mL of PPEF which is equivalent to 8XMIC of the sensitive *E. coli* K12 strain.

### Mutation determination by DNA sequencing of topoisomerase IA gene

Genomic DNA from the *de novo* generated resistant *E. coli* K12 mutants was isolated from 5 mL of overnight-grown culture using a QIAGEN kit according to the manufacturer’s protocol. Complete gene of *E. coli* topoisomerase IA was amplified using PCR. Amplified DNA products were resolved by electrophoresis on 1% agarose gels containing ethidium bromide. The polymerase chain reaction (PCR) products were purified using a QIAGEN gel extraction kit (Supplementary Fig. S3). Purified amplicons were processed for sequencing using an automated sequencer (Applied Biosystems; Lab India, Gurgaon, India). Untreated *E. coli* K12 sample were used as a control. Sequences were compared to check mutations using EMBOSS waters online software. The list of primers used for topoisomerase gene sequence is listed in (Supplementary Table S8) and were designed as per sequence reported previusly^34^.

### Quantification of gene expression using real-time quantitative PCR (qPCR)

Total RNA was extracted from the *E. coli* cells by using RNeasy Mini Kit (Qiagen). Reverse transcription was performed to yield cDNA using Accuscript cDNA synthesis kit (Agilent Technologies) with random primers following standard protocol. Target gene expression level was measured by quantitative PCR Brilliant II SYBR Green QPCR Master Kit (Agilent Technologies) on 7500 Real Time PCR System (Applied Biosystem). Relative gene expression was evaluated using the 2^−ΔΔCT^ method to calculate fold change in gene expression. Normalization of the transcriptional level was done comparing expression of the Glyceraldehyde 3-phosphate dehydrogenase (GAPDH) a house keeping gene as an endogenous control. Primers used in the study are same as the one reported previously^35^ and are listed in (Supplementary Table S9). Statistical significance analysis was done by student’s t-test and is defined as *p < 0.01.

### Bisbenzimide accumulation assay

The OD_600_ of all cultured suspensions were adjusted to 0.1 and 180 μL aliquots were seeded to each wells of Corning^®^ 96 Well Black with Clear Flat Bottom plate. 1 μM final concentration of PPEF was added to each well. Fluorescence was read from the top of the wells using excitation and emission filters of 340 and 475 nm, respectively, with 5 flashes/well; readings were taken for 30 cycles with a 60 s delay between each cycles using Tecan Infinite Pro 200 Reader^36^.

### PPEF uptake and cell death analysis by fluorescence microscopy

The cultures were treated with 1XMIC of PPEF and incubated at 37 °C for 1 h. 10^6^ cells were collected, washed and resuspended in 1 mL filtered PBS, pH 7.3, 1 μg PI was added per mL sample, and then incubated on ice for 30 min^37^. After incubation, cells were spotted onto glass slides and analyzed using a Zeiss microscope with 100x magnification, with axio software. Cells were analyzed using UV/488 nm dual excitation and emission was measured using standard 461 and 645 nm filters.

### Cell death analysis using flow cytometry

Cultures with ~10^6^ cells were treated with 1XMIC PPEF and incubated at 37 °C for 0, 5, 15, 30, 45 and 60 mins. At each time point, 50,000 events were recorded for PI uptake using Becton Dickinson LSR Fortessa^TM^ flow cytometer^38^.

### Checkerboard assay

Synergistic effect of combination of PPEF and CCCP was determined by Checker board method as described previously^39^. In the present study we have taken the combination as follows 1/2XMIC of CCCP fixed and two fold serial dilutions of PPEF similarly 1/2XMIC of PPEF fixed and two fold serial dilutions of CCCP. Minimal Inhibitory Concentration was determined using Tecan Micro-plate Reader at 600 nm.

The effect of combination was defined as per the FIC index, whereby FIC = FIC (PPEF) + FIC (CCCP), where FIC (PPEF) is the MIC of PPEF in the combination/MIC of PPEF alone, and FIC (CCCP) is the MIC of CCCP in the combination/MIC of CCCP alone.

Interpretation of FIC; antagonistic if FIC > 4.0, indifference if FIC > 1 and ≤4, additive if FIC > 0.5 and ≤1 and synergistic if FIC ≤ 0.5^40^,^41^.

### Time-kill assay

To evaluate the effect of PPEF, CCCP and combination of PPEF and CCCP on bacterial growth, a time-response growth curve was constructed according to the standards of the NCCLS^42^. 1 mL bacterial suspensions at a cell density of 10^7^ CFU mL^−1^ were exposed to PPEF (1XMIC), CCCP (0.5XMIC) and combination of PPEF (1XMIC) and CCCP (0.5XMIC). In the control tube equal volume of sterile miliQ water was added. These cultures were incubated at 37 °C with constant stirring at 200 rpm. Broth aliquots were collected at different time points, serially diluted in saline solution, plated on MH agar media and grown for 18 h at 37 °C to determine the total CFUs in each culture. Percentage cell recovered was calculated by dividing CFU calculated from treated over the CFU calculated from untreated cells at respective time points.

### Anti-Biofilm activity assay and cell viability count biofilm cells by MTT

As described earlier^43^ biofilm inhibition assays was performed by seeding 100 μL of bacterial suspension (~10^8^ CFU) into wells of 96 well plates in the presence of 1XMIC PPEF, 1XMIC CCCP, 1XMIC PPEF +1/2XMIC CCCP and 1XMIC CCCP +1/2XMIC PPEF for 24 h whereas, for preformed biofilm eradication assay, 100 μL bacterial suspensions (~10^8^ CFU) was first allowed to form biofilm for 24 h at 37 °C in static condition and then the formed biofilm was incubated with 1XMIC PPEF, 1/2XMIC CCCP, 1XMIC PPEF + 1/2XMIC CCCP for 24 h at 37 °C. In both the cases biofilm mass was evaluated by Crystal violet staining method.

Further, viable biofilm cells were determined by MTT assay^44^. The treatment conditions were same as described for biofilm inhibition assay and preformed biofilm inhibition assay. After treatment, the wells were washed with PBS and 200 μl of 0.5 mg/ml MTT in PBS was added to each well. After addition of MTT, the plate was incubated at 37 °C for 4 h followed by addition of 50 μl of 25% sodium dodecyl sulphate (SDS). Percentage viability was calculated with respect to control cells from the absorbance measurements at 595 nm.

### *In vitro* cytotoxicity Assay

The cell viability of HEK293T and NIH/3T3 cells against PPEF, CCCP and their combination were assessed by Clonogenic survival assay^45^. Both the cells were seeded at a density of 400 cells per well in a six-well flat bottom Corning^®^ costar^®^ cell culture plate. After 20 h, each compounds were added at 0.5, 2, 8 and 32 μg/mL concentrations. For combined treatment of PPEF and CCCP, 0.5 μg/ml and 2 μg/ml concentration of each were chosen. After subsequent treatment of 24 h, the drugs were removed, washed and the cells were allowed to grow further for 10 days to form colonies. The colonies were stained with 0.5% crystal violet and counted manually.

### Neutropenic Thigh Infection Model in Balb/c mice

Female Balb/c mice n = 6, per dosing group weighing 20–25 g were rendered neutropenic with 2 intraperitoneal injections of cyclophosphamide 150 mg/kg.bw and 100 mg/kg.bw on 4 days and 1 day prior to bacterial infection. 0.1 mL of the 10^6^ CFU/mL bacterial suspension was injected into right posterior thigh muscle. After 2 h post-infection mice were treated with PPEF (3 mg/kg.bw), CCCP (3 mg/kg.bw) and in combination PPEF + CCCP (3 mg/kg.bw + 3 mg/kg.bw) dissolved in 0.1 mL sterile water by single bolus intravenous injection. Twenty-four hours after antibacterial administration, the mice were humanely sacrificed. Right thigh muscles from each mouse were aseptically collected, homogenized and serially diluted and processed for quantitative cultures^46^,^47^.

### Statistical analysis

Data are expressed as means ± standard deviations for three independent experiments. Statistical analysis was done by One-way analysis of variance (ANOVA) followed by Tukey’s range test was applied for analysis of data with the level of significance set at p < 0.05.

## Additional Information

**How to cite this article**: Sinha, D. *et al*. Synergistic efficacy of Bisbenzimidazole and Carbonyl Cyanide 3-Chlorophenylhydrazone combination against MDR bacterial strains. *Sci. Rep.* **7**, 44419; doi: 10.1038/srep44419 (2017).

**Publisher's note:** Springer Nature remains neutral with regard to jurisdictional claims in published maps and institutional affiliations.

## Supplementary Material

Supplementary Information

## Figures and Tables

**Table 1:** Susceptibility of MDR Gram-negative clinical bacterial isolates against BBZs.

Strains	MIC (μg/mL) ± SD					
	PPVF	PYRVF	PYMVF	EPEF	PPEF	NNEF
***Acinetobacter baumannii***						
MTCC1920	128 ± 0.4	128 ± 0.6	128 ± 0.7	128 ± 0.6	128 ± 0.5	128 ± 0.9
AB387	32 ± 0.5	32 ± 0.4	16 ± 0.6	16 ± 0.1	8 ± 0.3	32 ± 0.4
AB312	32 ± 0.4	16 ± 0.2	32 ± 0.9	16 ± 0.1	4 ± 0.1	16 ± 0.2
***Klebsiella*** **spp**.						
*K. planticola* MTCC2272	128 ± 0.3	128 ± 0.7	128 ± 0.7	128 ± 0.8	128 ± 0.07	128 ± 0.8
*Klebsiella* sp. K1164	128 ± 0.4	128 ± 0.8	128 ± 0.6	128 ± 0.9	128 ± 0.8	128 ± 0.7
*Klebsiella* sp. K235	128 ± 0.3	128 ± 0.4	128 ± 0.9	128 ± 0.7	128 ± 0.4	128 ± 0.7
*Klebsiella* sp. K589	128 ± 0.6	128 ± 0.3	128 ± 0.7	128 ± 0.6	128 ± 0.6	128 ± 0.7
***Salmonella typhimurium***						
MTCC1251	8 ± 0.3	4 ± 0.1	16 ± 0.4	4 ± 0.1	0.25 ± 0.1	16 ± 0.7
ST412	0.25 ± 0.1	0.25 ± 0.1	0.25 ± 0.2	0.25 ± 0.1	0.25 ± 0.05	0.25 ± 0.0
***Pseudomonas aeruginosa***						
MTCC1688	128 ± 0.4	128 ± 0.5	128 ± 0.6	128 ± 0.7	128 ± 0.7	128 ± 0.9
PS162	16 ± 0.7	32 ± 0.7	16 ± 0.3	16 ± 0.6	8 ± 0.43	32 ± 0.2
PS366	128 ± 0.5	128 ± 0.9	128 ± 0.7	128 ± 0.8	128 ± 0.9	128 ± 0.7
***Providencia*** **spp**.						
MCC2102	128 ± 0.7	128 ± 0.7	128 ± 0.4	128 ± 0.6	128 ± 0.8	128 ± 0.9
P592	128 ± 0.3	128 ± 0.2	128 ± 0.9	128 ± 0.7	64 ± 0.5	128 ± 0.8
***Shigella flexineri***						
MTCC1457	8 ± 0.2	8 ± 0.4	16 ± 0.1	8 ± 0.06	2 ± 0.03	8 ± 0.3
***Enterobacter*** **spp**.						
MCC2289	128 ± 0.7	64 ± 0.8	64 ± 0.8	16 ± 0.3	16 ± 0.2	128 ± 0.9
E432	128 ± 0.6	128 ± 0.7	128 ± 0.8	16 ± 0.3	32 ± 0.5	64 ± 0.4
E589	128 ± 0.8	128 ± 0.6	128 ± 0.7	128 ± 0.5	32 ± 0.6	128 ± 0.8
E34	128 ± 0.8	128 ± 0.7	128 ± 0.7	16 ± 0.2	16 ± 0.4	64 ± 0.4

**Table 2:** Susceptibility of MDR Gram-positive bacterial isolates against BBZ.

Strains	MIC (μg/mL) ± SD					
	PPVF	PYRVF	PYMVF	EPEF	PPEF	NNEF
***Enterococcus*** **spp**.						
MCC2105	128 ± 0.7	128 ± 0.7	128 ± 0.9	128 ± 0.8	128 ± 0.9	128 ± 0.7
ENT1121	32 ± 0.04	32 ± 0.03	32 ± 0.02	16 ± 0.06	8 ± 0.02	16 ± 0.1
ENT1365	16 ± 0.07	32 ± 0.06	32 ± 0.02	16 ± 0.07	16 ± 0.06	32 ± 0.3
ENT1150	16 ± 0.09	16 ± 0.03	16 ± 0.2	32 ± 0.4	16 ± 0.5	32 ± 0.07
ENT1367	16 ± 0.3	16 ± 0.2	32 ± 0.1	16 ± 0.5	16 ± 0.7	16 ± 0.6
ENT439	16 ± 0.07	16 ± 0.65	16 ± 0.05	32 ± 0.03	8 ± 0.04	32 ± 0.6
***Staphylococcus*** **spp**.						
*S. aureus* MTCC740	8 ± 0.03	2 ± 0.02	128 ± 0.8	4 ± 0.03	2 ± 0.02	32 ± 0.4
MRSA ATCC43300	16 ± 0.3	1 ± 0.02	128 ± 0.7	4 ± 0.05	0.5 ± 0.01	16 ± 0.08
S976	32 ± 0.6	32 ± 0.05	32 ± 0.7	64 ± 0.2	16 ± 0.5	32 ± 0.5
S982	32 ± 0.5	32 ± 0.6	32 ± 0.07	16 ± 0.4	8 ± 0.07	32 ± 0.6
S1016	16 ± 0.08	16 ± 0.6	16 ± 0.5	16 ± 0.3	8 ± 0.1	32 ± 0.5

**Table 3:** Fractional Inhibitory Concentration Index of Bisbenzimidazole (PPEF) in combination against Efflux Pump Inhibitors CCCP.

Strains	PPEF (A) MIC μg/mL	CCCP (B) MIC μg/mL	(AB) MIC μg/mL	(BA) MIC μg/mL	FICI	Interpretation
***Escherichia coli***						
ATCC25922	16 ± 0.3	16 ± 0.3	2 ± 0.1	4 ± 0.1	0.37	Synergy
KK45	16 ± 0.6	16 ± 0.4	4 ± 0.1	1 ± 0.04	0.31	Synergy
***Acinetobacter baumannii***						
MTCC1920	128 ± 0.4	32 ± 0.5	32 ± 0.6	8 ± 0.3	0.5	Synergy
AB387	32 ± 0.5	16 ± 0.1	1 ± 0.1	2 ± 0.07	0.16	Synergy
***Klebsiella*** **spp**.						
MTCC 2272	128 ± 0.3	16 ± 0.2	32 ± 0.6	4 ± 0.2	0.5	Synergy
K 1164	128 ± 0.4	16 ± 0.4	32 ± 0.4	4 ± 0.1	0.5	Synergy
***Pseudomonas aeruginosa***						
MTCC1688	128 ± 0.4	>128	32 ± 0.5	32 ± 0.4	0.5	Synergy
PS 366	128 ± 0.5	>128	32 ± 0.7	32 ± 0.6	0.5	Synergy
***Enterococcus*** **spp**.						
MCC 2105	128 ± 0.9	32 ± 0.5	32 ± 0.6	8 ± 0.2	0.5	Synergy
ENT 1121	8 ± 0.2	16 ± 0.3	2 ± 0.2	4 ± 0.1	0.5	Synergy
***Providencia*** **spp**.						
MCC 2102	128 ± 0.8	64 ± 0.4	64 ± 0.7	16 ± 0.4	0.75	Additive
P592	64 ± 0.5	64 ± 0.3	32 ± 0.4	16 ± 0.6	0.75	Additive
