# Supplementary material for: Synergistic efficacy of Bisbenzimidazole and Carbonyl Cyanide 3-Chlorophenylhydrazone combination against MDR bacterial strains
Source: Sci Rep. 2017 Mar 17;7:44419. doi: 10.1038/srep44419 (PMC5355889; doi:10.1038/srep44419)
Supplement: Supplementary Information [file srep44419-s1.pdf]

Supplementary Information

Synergistic efficacy of Bisbenzimidazole and Carbonyl Cyanide 3-Chlorophenylhydrazone combination against MDR bacterial strains

Devapriya Sinha[1], Stuti Pandey[1], Raja Singh[2], Vinod Tiwari[1], Kirti Sad[2], VibhaTandon*[,1,2]

[1] Chemical Biology laboratory, Department of Chemistry, University of Delhi, Delhi, India

[2]Special Centre for Molecular Medicine, Jawaharlal Nehru University, New Delhi, India.

*To whom correspondence should be addressed

Special Centre for Molecular Medicine, Jawaharlal Nehru University, New Delhi-110067, India

Department of Chemistry, University of Delhi, Delhi-110007, India

Email: vtandon@mail.jnu.ac.in/vtandon@acbr.du.ac.in

Phone: 91-11-26742572, Fax: 91-11-26741781

Abbreviation used in Table S1, S2& S5

AMP: Ampicillin, KAN: Kanamycin, CIP: Ciprofloxacin, TET: Tetracycline; TRIM: Trimethoprim, CHL: Chloramphenicol, STR: Streptomycin; GEN: Gentamycin; NAL: Nalidixic acid; VAN: Vancomycin, CLI: Clindamycin; TSH: Co-trimoxazole; CTX: Cefotaxime; FOX: Cefoxitin; CPH: Ciprofloxacin; CL: Colistin;

Table S1. Minimum Inhibitory Concentration (µg/mL) (MIC) of antibiotics against Gram-positive and Gram-negative strains

Strain	AMP	KAN	CIP	TET	TRIM	CHL	STR	GEN	NAL	VAN
ATCC43300	32	128	0.5	0.25	128	8	8	32	---	32
MTCC740	64	8	0.25	0.25	>128	8	8	4	---	4
S1016	128	64	64	16	>128	8	64	0.25	2	>128
S976	1	0.25	1	8	1	1	0.5	0.25	1	>128
S982	2	64	16	8	128	4	2	16	---	---
MCC 2105	128	64	1	128	128	8	32	32	4	>128
Ent 1121	8	128	32	128	128	64	128	32	4	>128
Ent 1365	0.5	128	32	128	0.5	4	128	128	---	---
Ent 1150	2	64	1	128	0.5	0.5	32	8	---	---
Ent 1367										
ATCC25922	8	4	0.5	64	64	64	2	0.5	32	---
EC385	64	32	64	64	128	128	64	64	128	---
EC392	2	16	2	128	128	4	2	16	128	---
WB6	128	8	1	8	128	4	128	2	---	---
KK31	128	8	0.25	128	128	4	8	4	---	---
IPE	128	128	4	128	128	128	128	4	---	---
KK45	128	128	128	128	128	128	128	128	---	---
MTCC1920	16	8	4	16	16	8	0.25	4	4	---
Ab387	8	16	1	4	8	8	1	4	4	---
MTCC1688	32	32	1	16	64	8	8	4	32	---
Ps 162	32	16	4	4	16	8	0.25	64	32	---
Ps366	0.5	0.5	1	0.5	128	32	1	0.5	---	---
MTCC2272	32	16	16	4	16	8	128	4	16	---
K235	128	128	64	4	128	8	64	128	---	---
K589	128	128	32	8	128	128	128	128	---	---
K1164	32	64	32	16	16	16	32	64	32	---
MTCC 1457	8	16	1	4	8	8	0.25	4	16	---
MTCC 1251	32	64	4	16	16	8	4	16	32	---
St 412	64	64	16	64	8	16	0.25	4	32	---
MCC2102	1	8	0.25	64	1	128	64	4	---	---
P592	128	4	64	8	128	8	16	4	---	---
MCC2289	128	4	0.5	64	1	2	1	1	---	---
E 432	64	2	1	2	8	8	2	2	---	---
E589	128	128	32	128	128	128	64	128	---	---
E34	128	32	8	128	128	32	128	64	---	---

Table S2. Antibiotic Resistance profile of Gram negative/ Gram positive bacterial strains used in the study

Entry	Strains	Resistant to Antibiotics
1	MRSA ATCC43300	AMP, GEN, KAN, TMP, VAN
2	*S. aureus* MTCC740	AMP, TMP
3	*S. aureus* S1016	AMP, KAN, TET, CIP, TMP, VAN
4	*S. aureus* S976	VAN
5	*S. aureus* S982	GEN, KAN, CIP, TMP, VAN
6	*Enterococcus* sp. MCC 2105	AMP, GEN, KAN, TET, TMP, VAN
7	*Enterococcus* sp. Ent 1121	GEN, KAN, TET, CIP, TMP, CHL, VAN
8	*Enterococcus* sp. Ent 1365	GEN, KAN, TET, CIP
9	*Enterococcus* sp. Ent 1150	KAN, TET
10	*A. baumannii* MTCC1920	TET, TMP
11	*A. baumannii* Ab387	KAN
12	*P. aeruginosa* MTCC1688	AMP, KAN, TET, NAL, TMP
13	*P. aeruginosa* Ps 162	AMP, GEN, NAL, TMP
14	*P. aeruginosa* Ps366	TMP, CHL
15	*K. planticola* MTCC2272	AMP, CIP, TMP
16	*Klebsiella* sp. K235	AMP, GEN, KAN, CIP, TMP
17	*Klebsiella*sp K589	AMP, GEN, KAN, CIP, TMP, CHL
18	*Klebsiella*sp K1164	AMP, GEN, KAN, TET, CIP, NAL, TMP
19	*S. flexneri* MTCC 1457	---
20	*S. typhimurium* MTCC 1251	AMP, GEN, KAN, TET, CIP, NAL, TMP
21	*S. typhimurium* St 412	AMP, KAN, TET, CIP, NAL
22	*Providenci*a sp. MCC2102	TET, CHL
23	*Providenci*a sp. P592	AMP, CIP, TMP
24	*Enterobacter* sp. MCC2289	AMP, TET
25	*Enterobacter*sp E 432	AMP
26	*Enterobacter*sp E589	AMP, GEN, KAN, TET, CIP, TMP, CHL
27	*Enterobacter*sp E34	AMP, GEN, TET, CIP, TMP, CHL

Table S3. Minimal Bactericidal concentration (MBC) of BBZ against MDR Gram-negative clinical bacterial isolates

Strains	MBC (µg/mL)					
	PPVF	PYRVF	PYMVF	EPEF	PPEF	NNEF
MTCC 1920	>128	>128	>128	128	128	128
AB387	32	32	16	16	8	32
AB312	32	16	32	16	4	16
MTCC 2272	>128	>128	>128	128	128	128
K 1164	>128	>128	>128	128	128	>128
K 235	128	128	128	128	128	128
K 589	128	128	128	128	128	128
MTCC 1251	8	4	16	4	0.25	16
ST 412	0.25	0.25	0.25	0.25	0.25	0.25
MTCC 1688	>128	>128	>128	>128	128	128
PS 162	16	32	16	16	8	32
PS 366	128	128	128	128	128	128

MCC 2102	128	128	128	128	128	128
P 592	128	128	128	128	64	128
MTCC 1457	8	8	16	8	2	8

Table S4. Minimal Bactericidal concentration (MBC) of BBZ against MDR Gram-positive clinical bacterial isolates

Strains	MBC (µg/mL)					
	PPVF	PYRVF	PYMVF	EPEF	PPEF	NNEF
MCC 2105	128	128	128	128	128	128
ENT 1121	32	32	32	16	8	16
ENT 1365	16	32	32	16	16	32
ENT 1150	16	16	16	32	16	32
ENT 1367	16	16	32	16	16	16
ENT 432	16	16	16	32	8	32
MTCC 740	8	2	128	4	2	32
ATCC 43300	16	1	128	4	0.5	16
SA 976	32	32	32	64	16	32
SA982	32	32	32	16	8	32
SA 1016	16	16	16	16	8	32

Table S5. MIC values in µg/mLof different antibiotics against susceptible *E. coli* K-12 (S) and *de novo* generated resistant *E. coli* K-12 (R) against PPEF

Strain	CHL	TET	CLI	TSH	CTX	FOX	CPH	CL	AMP	KAN	PPEF
K-12 (S)	6	2	6	0.19	0.125	12	0.094	1.5	3	1.5	8
K-12 (R)	12	4	12	0.25	0.25	32	0.125	1.5	6	1.5	64

Table S6. MBC values in µg/mL of PPEF against clinical *E. coli*strains used in the study.

Entry	KK45	WB6	IPE	KK31	EC385	EC392
PPEF	16	8	8	4	8	8

Clinical *E. coli* isolates obtained from IOP Safdarjung Hospital, New Delhi, India

Table S7. Susceptibility of Efflux Components, Porin, deficient *E. coli* K12 Mutants against PPEF

MBC(µg/mL)							
Entry	*E. coli K12*	Δ*acrA*	Δ*emrA*	Δ*tolC*	Δ*ompC*	Δ*ompF*	K12(R)
PPEF	8	2	4	1	8	8	64

E. coli K12 derived engineered deleted mutants Δ*acrA*, Δ*emrA*, Δ*tolC*, Δ*ompC*, Δ*ompF*were obtained from Yale CGSC, The Coli Genetic Stock Center.

Table S8. Primers used for Topoisomerase IA Gene Sequencing

Forward primer	
1.	TACGCTGCGTGACGCCGGTG
2.	TATAGCGCCTGTAGGCCAAG
3.	CAGCTGCCAAAAAGAGTGCC
4.	ACAAACCGGGTGAGCTGAA
5.	CCAGCACCCGTCTTGGATTT
Reverse Primers	
1.	TTGCCAGATTGACGAAAGCG
2.	CAATTGTGCCACGGGTCAAC
3.	CTGTAGCGCGCTTTTTCCAG

Table S9. List of Primers used in real-time study

Gene	Forward primer	Reverse primer
acrA	5’ CTTAGCCCTAACAGGATGTG 3’	5’ TTGAAATTACGCTTCAGGAT3’
acrB	5’ CGTACACAGAAAGTGCTCAA3’	5’ CGCTTCAACTTTGTTTTCTT3'
emrA	5’ CAACCGGTAAAGAAGAGG3’	5’ GTTATCGGCCCAGACTTTG3’
emrB	5’ ATTATGTATGCCGTCTGCTT3’	5’ TTCGCGTAAAGTTAGAGAG3’
mdfA	5’ TTTATGCTTTCGGTATTGGT3’	5’ GAGATTAAACAGTCCGTTGC3’
ompC	5’ CTTCAAAGGTGAAACTCAGG3’	5’ GTTGTCAGAACCGTAGGTGT3’
ompF	5’ GAACTTCGCTGTTCAGTACC3’	5’ CGTACTTCAGACCAGTAGCC3’
rob	5’ GTCGTCTTTATCCTGACTCG3’	5’ TTTGTCACCCTGGAAGATAC3’
tolC	5' AAGCCGAAAAACGCAACCT 3'	5' CAGAGTCGGTAAGTGACCATC 3'
yhiV	5’ GCACTCTATGAGAGCTGGTC3’	5’ CCTTCTTTCTGCATCATCTC3’
gapdh	5' ACTTACGAGCAGATCAAAGC3'	5' AGTTTCACGAAGTTGTCGTT3'

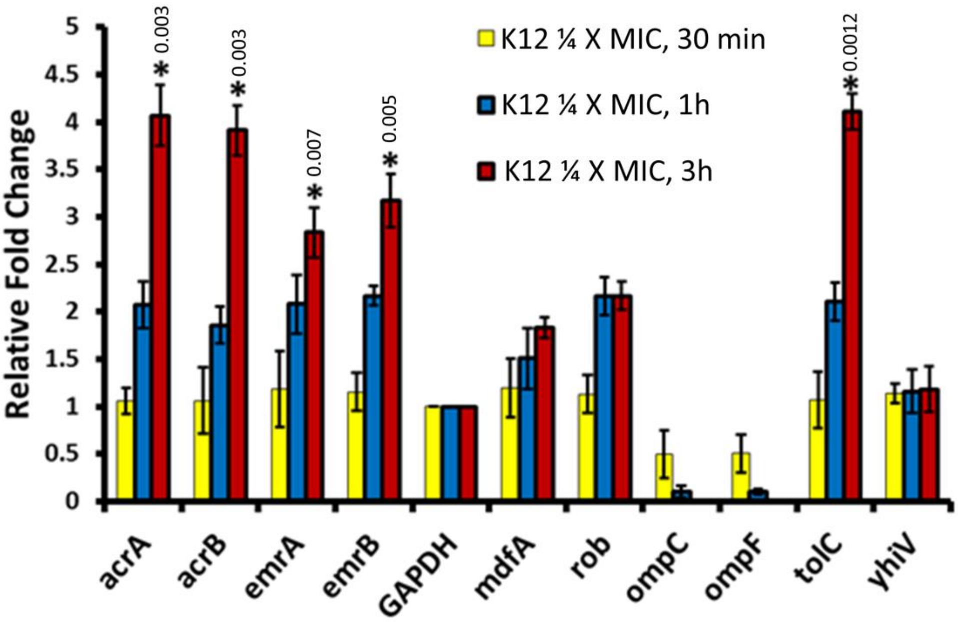

Figure S1. Relative fold change in Gene Expression of *E. coli* K12 treated with ¼XMIC Ciprofloxacin for 30mins, 1h and 3h. Results are presented of three independent experiments (n=3) presented as mean ± SD. Statistical comparison between control and experimental conditions were found to be statistically significant with * $p<0.01$. Detailed statistically significant p-values of control *vs.* experimental results are indicated.

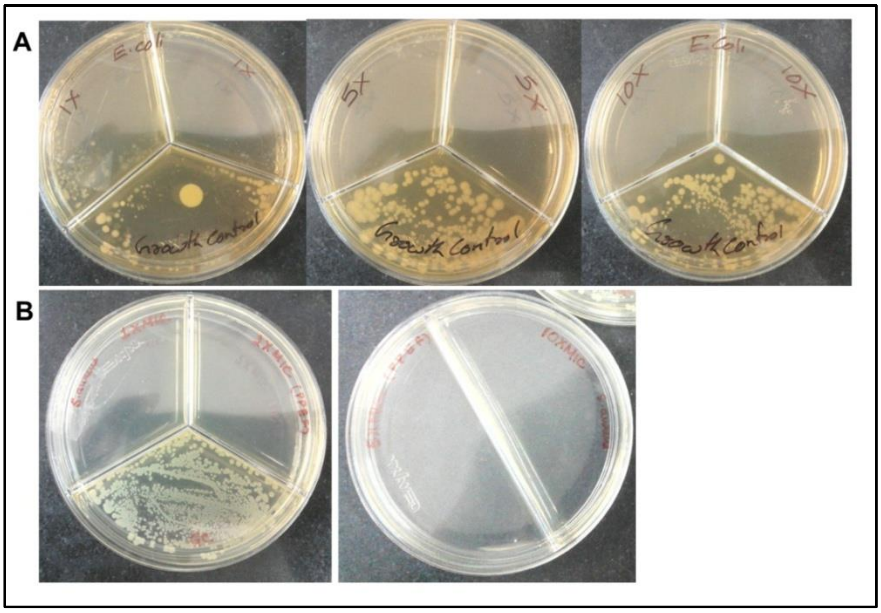

Figure S2. Spontaneous mutation generation against PPEF. (A) *E. coli* K12 10^9cells treated with 1X, 5X and 10X MIC of PPEF for 48h. In Growth control the bacterial culture was diluted in 10^{6-7} and then plated to get countable colonies. (B)*S. aureus* MTCC7401$0^9$cells treated with 1X,2X, 5X and 10X MIC of PPEF for 48h. In Growth control the bacterial culture was diluted in 10^{6-7} and then plated to get countable colonies.

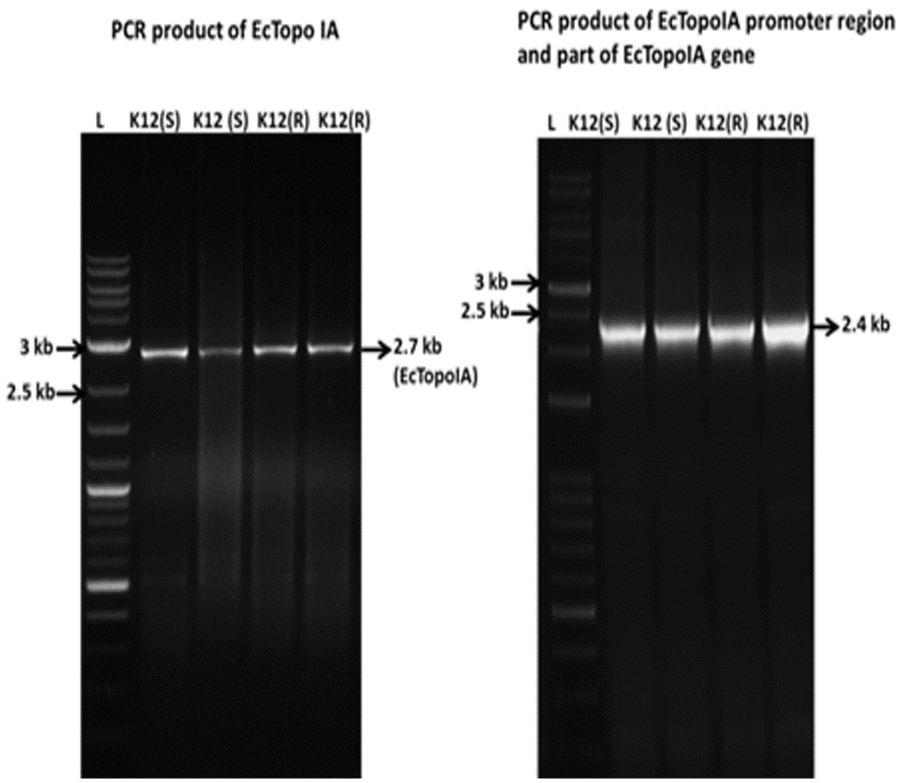

Figure S3. PCR amplified product of *E. coli* Topoisomerase IA gene and the upstreram promoter region

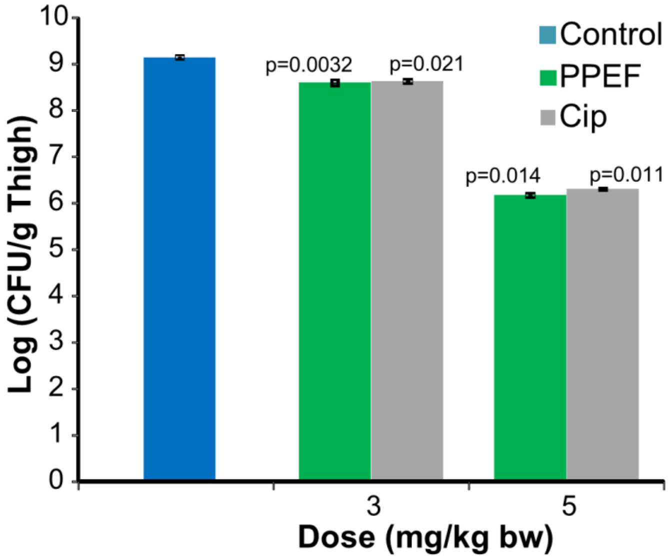

Figure S4. Efficacy of PPEF and ciprofloxacin (Cip) in neutropenic thigh infection model. Bar graph designate $\log_{10}$ (CFU/g thigh) *vs* drug dose. The error bars represent results of mean ± SD of (n=6) mice/group.

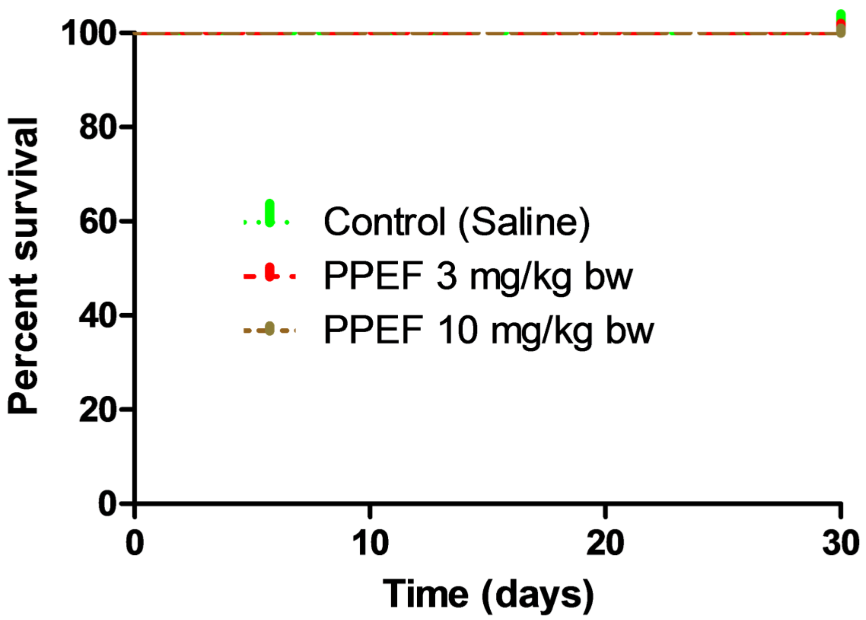

Figure S5. ***In vivo* survival study against PPEF.** 30 day Balb/c mice survival was studied against 3 and 10 mg/kg PPEF treatment through intra-venous mode of administration. Control mice were administered intravenously equal amount of saline and observed for survival (n=6/group).
